# Transitions of motor neuron activities during *Ciona* development

**DOI:** 10.3389/fcell.2023.1100887

**Published:** 2023-01-13

**Authors:** Madoka K. Utsumi, Kotaro Oka, Kohji Hotta

**Affiliations:** ^1^ Department of Biosciences and Informatics, Faculty of Science and Technology, Keio University, Yokohama, Japan; ^2^ Waseda Research Institute for Science and Engineering, Waseda University, Shinjuku, Japan; ^3^ Graduate Institute of Medicine, College of Medicine, Kaohsiung Medical University, Kaohsiung, Taiwan

**Keywords:** central pattern genenator, *Ciona intestinalis* type A (*Ciona robusta*), half center model, locomotion, motor circuit development, Ca^2+^ imaging, optogenetics, motor neuron

## Abstract

Motor neurons (MNs) are one of the most important components of Central Pattern Generators (CPG) in vertebrates (Brown, Proceedings of The Royal Society B: Biological Sciences (The Royal Society), 1911, 84(572), 308–319). However, it is unclear how the neural activities of these components develop during their embryogenesis. Our previous study revealed that in *Ciona robusta* (*Ciona intestinalis* type A), a model organism with a simple neural circuit, a single pair of MNs (MN2L/MN2R) was determining the rhythm of its spontaneous early motor behavior (developmental stage St.22-24). MN2s are known to be one of the main components of *Ciona* CPG, though the neural activities of MN2s in the later larval period (St.25-) were not yet investigated. In this study, we investigated the neural activities of MN2s during their later stages and how they are related to *Ciona*’s swimming CPG. Long-term simultaneous Ca^2+^ imaging of both MN2s with GCaMP6s/f (St.22-34) revealed that MN2s continued to determine the rhythm of motor behavior even in their later larval stages. Their activities were classified into seven phases (I-VII) depending on the interval and the synchronicity of MN2L and MN2R Ca^2+^ transients. Initially, each MN2 oscillates sporadically (I). As they develop into swimming larvae, they gradually oscillate at a constant interval (II-III), then start to synchronize (IV) and fully synchronize (V). Intervals become longer (VI) and sporadic again during the tail aggression period (VII). Interestingly, 76% of the embryos started to oscillate from MN2R. In addition, independent photostimulations on left and right MN2s were conducted. This is the first report of the live imaging of neural activities in *Ciona*’s developing swimming CPG. These findings will help to understand the development of motor neuron circuits in chordate animals.

## Introduction

Central Pattern Generator (CPG) is a locomotor network of neurons distributed along the vertebrate spinal cord, which generates rhythmically patterned outputs in the absence of rhythmic inputs (Grillner and Kozlov, 2021). CPG is necessary for controlling rhythmic activities such as walking, swimming, chewing, and breathing. However, it is not yet clear how these rhythmic neural activities of chordate locomotion are generated during their embryogenesis. In 1911, a hypothesis for the pattern-generating mechanisms of CPG was introduced as the “Half Center Model” (Brown, 1911). According to this model, CPG consists of flexor and extensor motor neurons, interneurons, and contralateral inhibitory neurons. When the extensor side is stimulated and its ipsilateral muscle contracts, an inhibitory signal is transported to the contralateral flexor side. This suppresses the flexor muscle’s contraction while the extensor muscle is being contracted. During the repetition of such reciprocal contralateral inhibition, flexor muscles and extensor muscles will be contracted alternately and, consequently, generate a rhythmical locomotory pattern.

The mechanisms for generating rhythmic neuronal bursts during swimming behaviors are already well-studied with CPG of lampreys. In the case of lampreys, it was revealed that the swimming-related burst generators are distributed along their spinal cords, inhibitory neurons are involved in pattern generation, and their contralaterally extending neurons are GABAergic (Biró et al., 2008; Lacalli and Candiani, 2017; Grillner and Kozlov, 2021). In zebrafish, motor neurons are the first observed oscillating neurons during its motor circuit development (Wan et al., 2019). However, even in vertebrates, how CPG develops during embryogenesis is not yet fully understood due to the untraceability of neuronal activities from early developmental stages and the complexity of their neuronal networks.

Because ascidians are vertebrates’ closest relatives (Satoh et al., 2014), they are studied intensively in the fields of chordate evolution and developmental biology. In addition, the cell lineage and neural connectome of *Ciona* larvae are already revealed (Horie et al., 2008; 2009; Ryan et al., 2016; Gibboney et al., 2020; Kim et al., 2020; Borba et al., 2021; Kourakis et al., 2021; Olivo et al., 2021). Their larval neural network consists of only <330 neurons, and their CNS exhibits anatomical asymmetry (Kourakis et al., 2021). These unique and simple characteristics of *Ciona* enable us to study their neural activity during embryonic development at a single-cell level. For instance, in peripheral nervous systems, recent research has found that two isoforms of the RIM-binding proteins (Rimbps) family, one of the crucial components of the presynaptic *active zone* and Ca^2+^ homeostasis in flies and humans, were also expressed in *Ciona* (Coppola et al., 2020).

**FIGURE 1:**
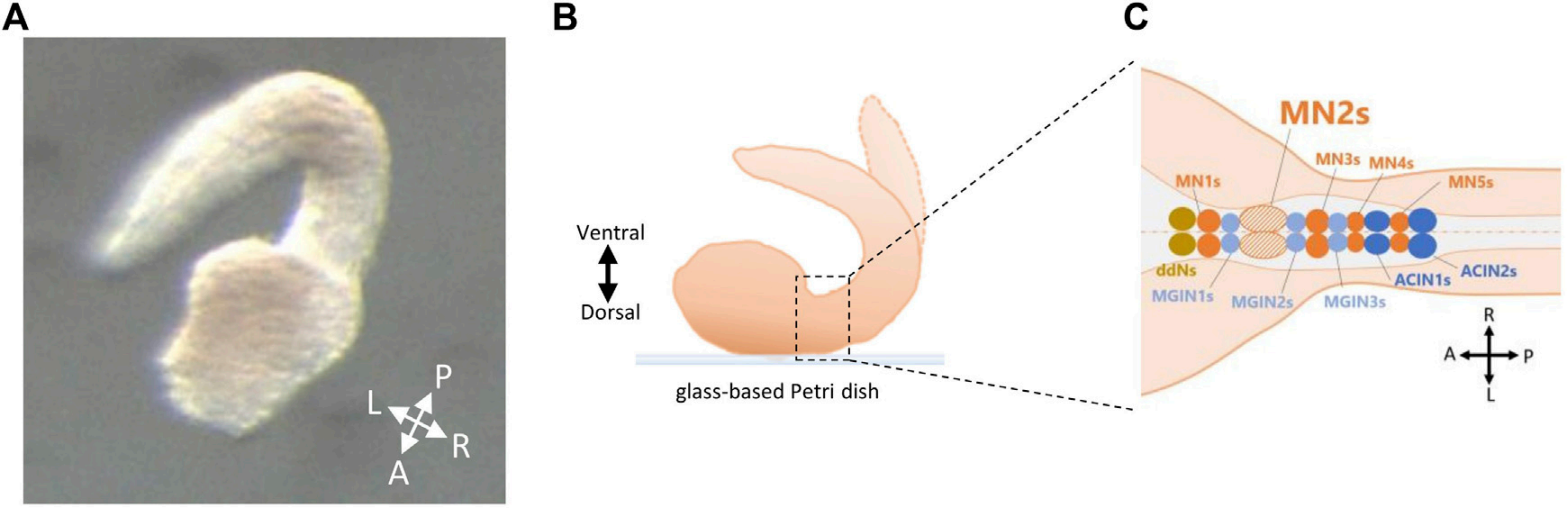
Fixation of *Ciona* embryos on glass-based Petri dish for MN2L and MN2R imaging. **(A)** Larvae fixed on a glass-based Petri dish by its dorsal side (method used in this study). A, anterior; P, posterior; L, left; R, right. **(B)** Schematic illustration of a fixed larva on a glass-based Petri dish by its dorsal side. **(C)** Neurons located in the motor ganglion (MG) region of *Ciona* swimming larva seen from its dorsal side. ddNs, descending decussating neurons; MN1-5s, motor neuron 1-5s; MGIN1-3s, motor ganglion interneurons 1-3s; ACIN1-2s, ascending contralateral inhibitory neurons 1-2.

Though adult *Ciona* is sessile, their hatched tadpole-shaped larvae can swim *via* their somatic muscles, distributed on the left and right sides of their tails (Nishino et al., 2011). Such swimming behavior is considered to be generated mainly by five pairs of neurons (descending decussating Neuron, A12.239/ddN; Motor Ganglion Interneuron 1, A13.474/MGIN1; Motor Neuron 1, A11.118/MN1; Motor Ganglion Interneuron 2, A11.117/MGIN2; Motor Neuron 2, A10.64/MN2) in their Motor Ganglion (MG) region, and two pairs of Ascending Contralateral Inhibitory Neurons (ACIN1, ACIN2) (Figure 1C) (Horie et al., 2010; Nishino et al., 2011; Ryan et al., 2016). However, the mechanisms behind the generation of *Ciona* swimming larva were a mystery. For instance, the descending decussating neurons (ddNs), which are considered to be homologous with Mauthner cells, project axons contralaterally to MN2 (Ryan et al., 2017). There is a possibility that ddNs may play an important role in tail flicking movements. In addition, Ca^2+^ signaling activity of the non-reproductive Gnrh2-expressing cells (Okawa et al., 2020) is thought to correlate with its swimming behavior.

Recent research (Hara et al., 2022) revealed that the trunk-tail junctional region in *Ciona* larvae, which corresponds with the MG region, autonomously expresses periodic tail-beating bursts at constant intervals (-20 s).

A previous study (Akahoshi et al., 2021) revealed that a single pair of motor neurons, A10.64/MN2, determines the rhythm of early motor behavior in *Ciona.* MN2 neurons show Ca^2+^ oscillation with an -80 s interval in the early tailbud stage (-St.24). This activity was cell-autonomous, because MN2 could oscillate even at a dissociated single-cell level. This Ca^2+^ oscillation coincides with unilateral tail muscle contracting behaviors observed in tailbud stages (Early Tail Flicks, ETFs). In addition, the bursts of the action potential in MN2 gradually become tuned to the rhythmic ipsilateral muscle contractions occurring in each ETF (Akahoshi et al., 2017; Akahoshi et al., 2021).

The purpose of this study is to uncover the neural activities of MN2s in later developmental stages. When compared to Brown’s Half Center Model, MN2s could be estimated to be one of the main components of *Ciona* CPG. In this study, a method to fix larvae on their dorsal sides was invented. With this new experimental system, long-term simultaneous imaging of both left and right MN2s after St.22 (-40 h) and independent photostimulations on each MN2 were conducted for the first time. This enabled us to compare the differences between MN2L and MN2R activities and their synchronicity. This is the first report of the live imaging of neural activities in *Ciona*’s developing motor circuit, which is assumed to be its swimming CPG.

## Materials and Methods

### Cultivation of adult *Ciona*

*Ciona robusta* (*Ciona intestinalis* type A) adults (wild type) (Pennati et al., 2015) were regularly obtained from Misaki Marine Biological Station (The University of Tokyo), Maizuru Fisheries Research Station (Kyoto University), and Onagawa Field Center (Tohoku University) through the National Bio-Resource Project (NBRP).

### Electroporation

Eggs collected in seawater were fertilized and kept at room temperature for 30 min. At 30 min post-fertilization, eggs were centrifuged, and the seawater was completely replaced with 0.77 M mannitol in 10% seawater (21303-405, Nacalai Tesque). Plasmid-construct (around 600–1000 ng/μl) was combined with the egg containing 0.77 M mannitol mixture. Electroporation was performed according to the previous study (Hirai et al., 2017). In the case of electroporating two constructs, 20 µl of each plasmid-construct was applied. 400 μl of this solution was placed in a 2 mm cuvette. Electroporation was performed using the Gene Pulser Xcell Electroporation System with Capacitance Extender (Bio-Rad, Hercules, CA). The settings for the machine were 15 V/cm, and the time constant of the pulse was 25 mseconds.

### Fixation of the dorsal side of the embryo

In this study, a method to fix *Ciona* embryos by their dorsal side was invented. The methods were as follows. Embryos were collected by a glass pipette and fixed on a glass-based Petri dish (glass diameter φ12 mm or φ27 mm, IWAKI, Shizuoka, Japan). The direction of the embryos was modified by making a weak water stream with glass pipettes or by gently touching the embryos with a pulled glass needle. When the larvae started to swim, micro cover glass (φ18 mm, MATSUNAMI, Osaka, Japan) was placed on the embryos and fixed on the glass-based dish with grease (Figures 1A, B).

### Live imaging by 3CCD camera

**FIGURE 2:**
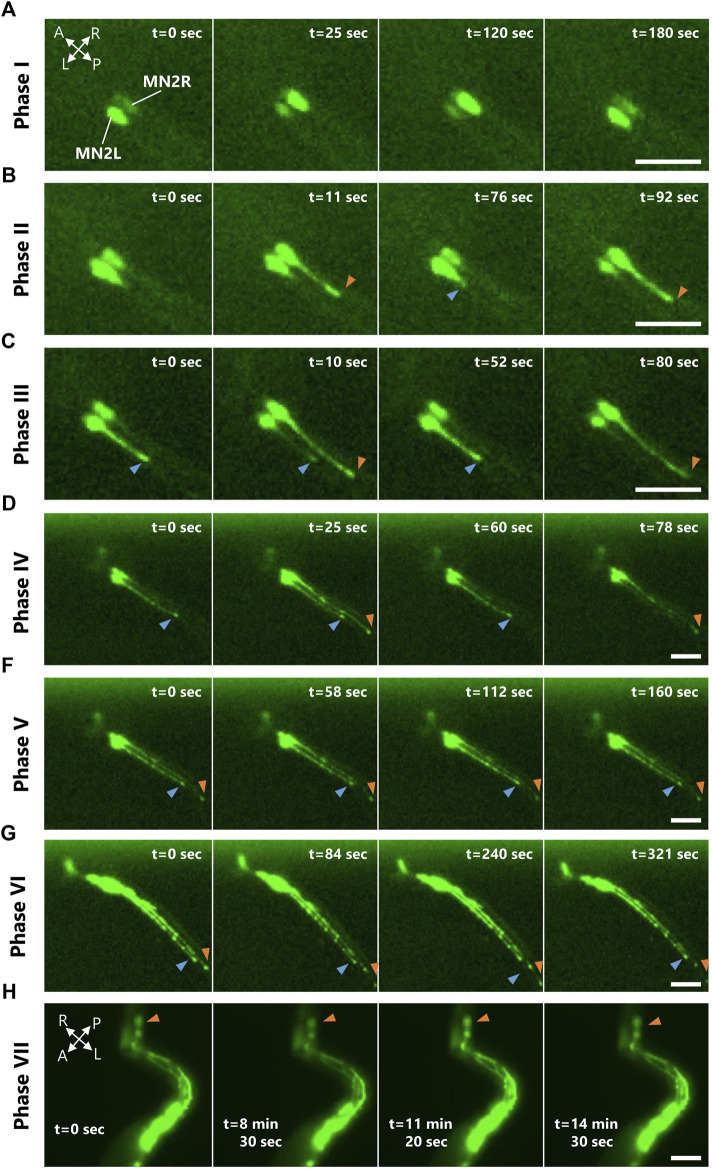
MN2L and MN2R Ca^2+^ transients observed from tailbud stage to tail absorption. Representative images of the four consecutive Ca^2+^ bursts occurring in either MN2L, MN2R, or both in each Ca^2+^ phase observed with pSP-CiVAChT:GCaMP6s transduced larva. Images taken by fluorescent microscopy. A, anterior; P, posterior; R, right; L, left. Blue/orange arrowheads indicate MN2L/MN2R axon terminals. Scale bar 50 µm. **(A)** Phase I. **(B)** Phase Ⅱ. **(C)** Phase Ⅲ. **(D)** Phase IV. **(E)** Phase V. **(F)** Phase VI. **(G)** Phase VII.

**FIGURE 3:**
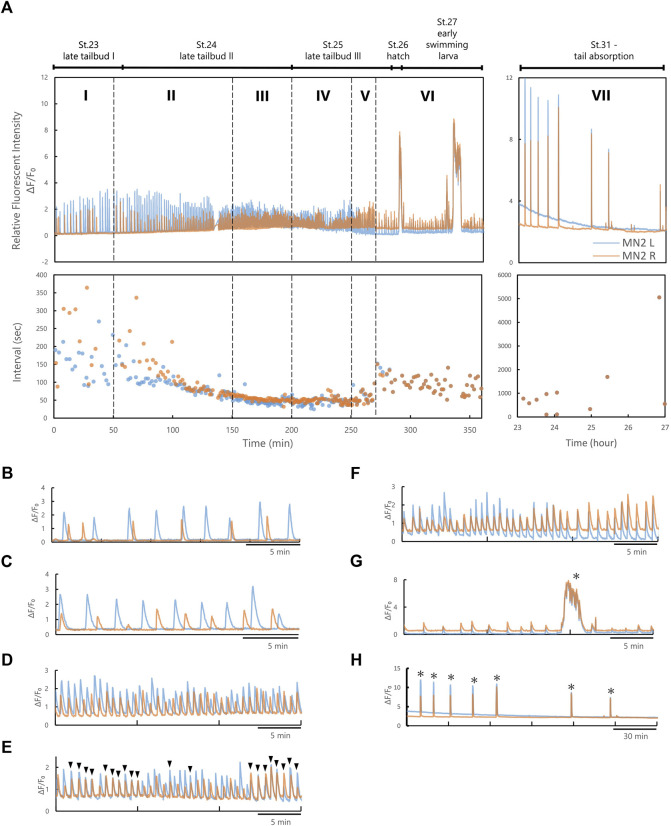
MN2L and MN2R Ca^2+^ transients could be divided into seven phases based on their synchronicity and inter-burst-intervals. **(A)** An example of the Relative Fluorescent Intensity (RFI) and intervals of Ca^2+^ transients observed with pSP-CiVAChT:GCaMP6s transduced larva (temperature, 0-9.5 hpf 22°C, during imaging 7.5 h 20°C). Blue, MN2L; orange, MN2R. **(B)** Phase I. **(C)** Phase Ⅱ. **(D)** Phase Ⅲ. **(E)** Phase IV. **(F)** Phase V. **(G)** Phase VI. **(H)** Phase VII. Black arrowheads represent the timing when the Ca^2+^ peak of MN2L and MN2R synchronized, and asterisks represent Ca^2+^ bursts.

The fixed *Ciona* larvae on a glass-based dish were observed by fluorescence microscopy with a 3CCD camera. A long-term Ca^2+^ transient was captured with pSP-CiVAChT:GCaMP6s or pSP-CiVAChT-H2B:GCaMP6s transduced *Ciona* embryos (N = 10, Figure 2; Figure 3., Supplementary Table S1). The same results can be obtained with either pSP-CiVAChT:GCaMP6s or pSP-CiVAChT-H2B:GCaMP6s (data not shown). An inverted microscope (Nikon Eclipse, IX71) with a 10×, 20×, 60× oil immersion objective lens (LUCPlanFLN) was used for fluorescence imaging with a U-MWBV2 mirror unit (Olympus, Shinjuku, Japan). SOLA LED light (Lumencor, Beaverton, OR) was used as a light source; fluorescence images were acquired with a 3CCD camera (C7800-20, Hamamatsu Photonics, Hamamatsu, Japan) and processed by the AQUACOSMOS software (Hamamatsu Photonics). Room temperature was maintained at 20°C. As a result, Ca^2+^ transients for both MN2L and MN2R, located at the *Ciona* motor ganglion region (Figure 1C), were continuously imaged from St.22 (mid tailbud II) to St.34 (late tail absorption) (N = 10, Supplementary Table S1). *Ciona* developmental staging (Hotta et al., 2007) was used to estimate the developmental stage from morphology and time after fertilization.

### Live imaging by CLSM

*Ciona* larvae fixed on a glass-based dish were observed by confocal laser-scanning microscopy (CLSM). An Olympus fv1000 microscope was used, and the images were processed by an Olympus fv1000 viewer. Excitation was performed at 488-nm to visualize GCaMP and photostimulate ChR2, and 559-nm to visualize mCherry. Olympus 20× or 40× oil immersion lenses were used. Room temperature was maintained at 20°C. Laser power density was measured by the optical power meter 3664 (HIOKI, Ueda, Japan) and optical sensor 9742 (HIOKI).

### Single-cell photostimulation of MN2

Single-cell photostimulation of MN2 after St.27 (swimming larva) was performed with Channelrhodopsin-2 (hChR2; reference (Zhang et al., 2007) transduced larva. pSP-Neurog-hChR2(E123T/T159C):mCherry were electroporated to express ChR2 at MN2L and MN2R. Laser irradiation was performed by a CLSM Olympus fv1000 microscope. After St.24, pSP-Neurog-hChR2(E123T/T159C):mCherry was expressed exclusively in the prRNs, MN2s, MN1s, and MGIN1s (depending on the larva due to mosaic expression). Therefore, to avoid photostimulating neurons other than MN2s, the region of interest (ROI) for laser irradiation was restricted to the targeted neuron. Stimulation was performed by irradiation with a 488 nm laser for 0.3 s (laser power density 15 μW/cm^2^).

### Live imaging with a high-speed camera and tail curvature quantification

The swimming behavior of *Ciona* larvae was recorded with a high-speed camera (WRAYCAM-VEX230M, WRAYMER, Osaka, Japan) attached to a stereoscopic microscope (SZX12, Olympus). Room temperature was kept at 20°C. Images were captured at 200–400 frames per second (fps). Tail curvature was quantified from these images.

To quantify the tail movement of *Ciona* swimming behavior, images obtained by high-speed camera were processed with the open-source programs Fiji (National Institutes of Health, Bethesda, MD) and MATLAB (MathWorks). Image denoising, binarization, and skeletonization were processed by Fiji and exported as a .BMP image sequence. Skeleton pruning was processed in MATLAB. The midline of the larva was extracted, and the curvature of the midline was calculated with the position of three points on the midline: start-(A), mid-(B), and end-(C) (Akahoshi et al., 2021). The equations used to calculate the curvature were as follows:

$$curv.=\frac{1}{R}=\frac{2\sin\;C\;}{C}=\frac{2}{\left|\vec{A-B}\right|}\frac{\left(\vec{A-C}\right)\times \left(\vec{B-C}\right)}{\left|\vec{A-C}\right|\left|\vec{B-C}\right|}$$

### Calculation of Ca^2+^ peaks, intervals, and durations

Fluorescent intensity was detected by the AQUACOSMOS software (Hamamatsu Photonics). Data were analyzed with MATLAB (MathWorks, Natick, MA) and Excel (Microsoft, Redmond, WA). The fluorescent intensity data of each ROI (Supplementary Figures S1A, B) was imported to and processed with MATLAB. The time course of Relative Fluorescent Intensity (RFI; ΔF/F₀) was normalized, differentiated, and filtered with a Savitzky-Golay filter (Supplementary Figure S1C). Peak detection with this denoised differentiated data was done with the MATLAB Signal Processing ToolboxTM package. Burst intervals and durations were calculated as follows. Intervals were defined as the time between each peak and duration was defined as the time from when the denoised differentiated intensity started to elevate from 0 (A.U.) before each peak to when the intensity elevated to 0 (A.U.) after each peak (Supplementary Figure S1D).

### Circular statistics (Rayleigh test)

**FIGURE 4:**
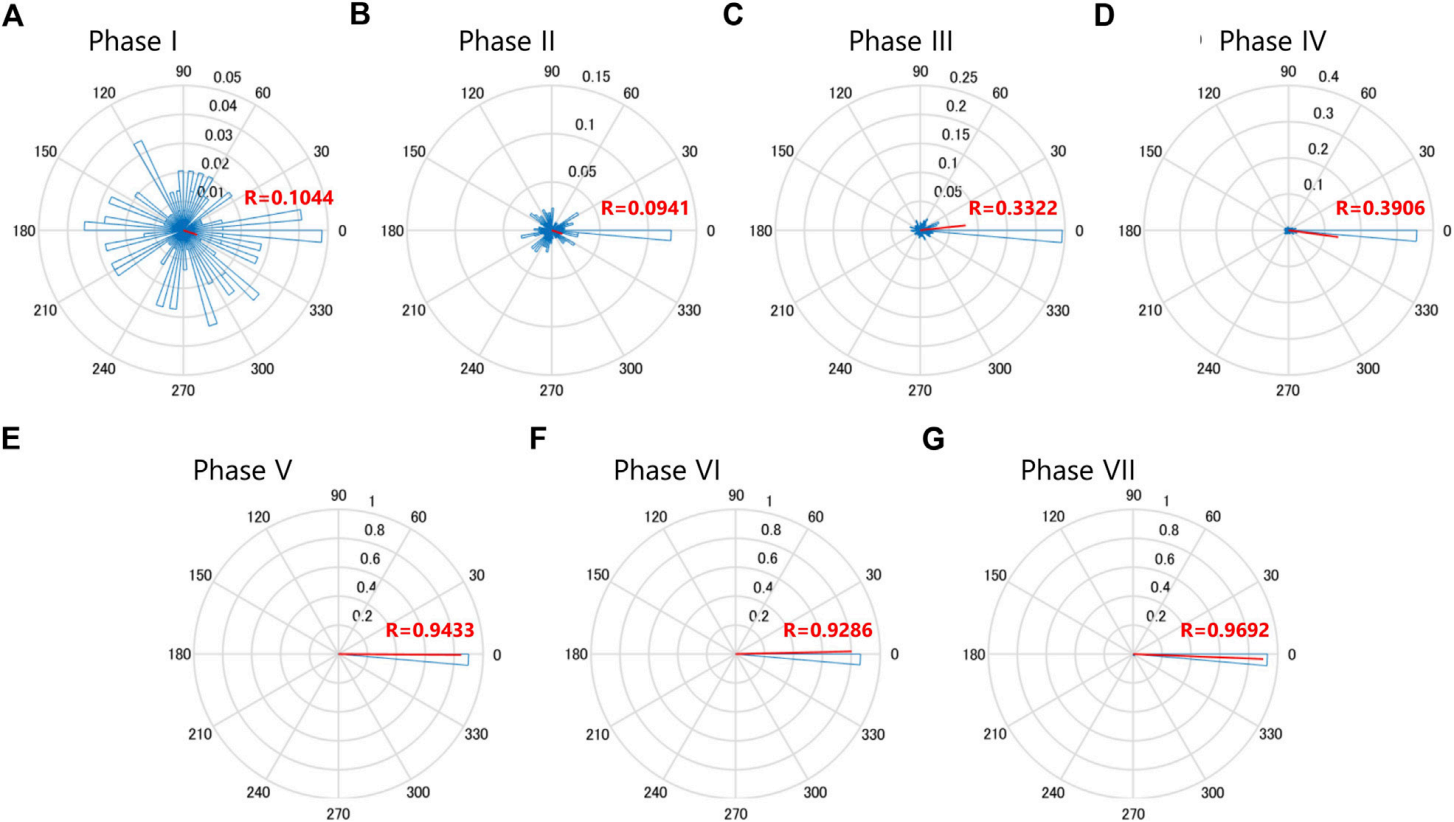
MN2L and MN2R Ca^2+^ transients gradually synchronize as they develop into swimming larvae. Rayleigh’s test of uniformity for the MN2L and MN2R Ca^2+^ transients. Time point of the MN2R peak is assigned as a phase value of 0°, and that of the next MN2R is assigned as a phase value of 360°. The Phase values of MN2L Ca^2+^ burst peak timings are plotted as the blue histograms, and the average values are shown as the vector (red lines). The direction of the vector shows the mean of the MN2L Phase value, whereas the length of the vector (0≦R≦1) shows the uniformity of MN2L burst Phase values. **(A)** Phase I, mean -18.3°, R = 0.104, n = 145. **(B)** Phase Ⅱ, mean -18.4°, R = 0.0941, n = 59. **(C)** Phase Ⅲ, mean 6.17°, R = 0.332, n = 132. **(D)** Phase IV, mean -7.78°, R = 0.391, n = 334. **(E)** Phase V, mean -0.359°, R = 0.943, n = 49. **(F)** Phase VI, mean 1.27°, R = 0.929, n = 99. **(G)** Phase VII, mean -3.00°, R = 0.969, n = 112.

For the phase analysis of Ca^2+^ burst timings of MN2L and MN2R, Rayleigh tests (Zar, 1999) were conducted with the MATLAB Circular Statistic package (https://github.com/circstat/circstat-matlab). The time point of the MN2R Ca^2+^ peak was assigned as the phase value of 0°, while that of the next MN2R Ca^2+^ peak was assigned as the phase value of 360°. Vector (Figure 4, red) shows the mean of the MN2L phase value, whereas the length of the vector (0≦R≦1) shows the uniformity of MN2L burst phase values.

### Tracking tail movements with DeepLabCut

As for the fluorescence microscopic images, tail movements were automatically tracked with the open-source program DeepLabCut, a software package for animal pose estimation (version 2.2, https://deeplabcut.github.io/DeepLabCut/docs/intro.html; (Nath et al., 2019)). Twenty image data were extracted randomly from the analysis video, and then three body parts on the larva (“bodypart1”, ocellus; “bodypart2”, middle of the tail; “bodypart3”, terminal of the tail) were labeled manually for use as a training dataset. Training data sets were prepared individually from each Phases. Training was conducted with a maximum of 50,000 iterations. Trajectories of the three body parts were exported to an Excel file, and tail bending angle (°) was calculated from these three coordinates.

## Results

### Activities of left and right MN2s (St.22 - St.34) classified into seven phases

To capture images of both left and right MN2s using an inverted microscope, *Ciona* embryos were fixed on a Petri dish on their dorsal side. However, in previous studies most of the embryos were captured from the lateral side, or studies used embedded larvae for live imaging (Okawa et al., 2020; Kolar et al., 2021). Therefore, in this study, we developed a protocol to fix *Ciona* embryos on their dorsal side (see Materials and Methods). This method enabled live-imaging of both MN2L and MN2R without restricting its natural embryonic development or tail muscle movement.

As a result, Ca^2+^ oscillations at both MN2L and MN2R were measured even after St.24 (Supplementary Video S1). The Ca^2+^ burst durations and the burst intervals of Ca^2+^ transients between MN2L and MN2R changed as they developed from the tailbud, swimming larva, adhesion, and tail absorption stages (Figure 2). There were also individual differences in which left or right MN2 started to oscillate earlier than the other. Interestingly, 76% of the embryos started to oscillate from MN2R, meaning that MN2R was likely to be the dominant oscillating motor neuron as compared to MN2L (N = 13/17). Relevant to this finding was that the extent of the axon growth was asymmetric. MN2R with a longer elongated axon oscillated earlier than the other MN2L (Figure 2, blue and orange arrowheads, Supplementary Video S2).

From the images captured with fluorescent microscopy, the time-dependent Relative Fluorescent Intensity (RFI) change, Ca^2+^ elevation peak timing, and Ca^2+^ burst intervals (= intervals) were calculated (Figure 3A; Supplementary Figure S1). Ca^2+^ oscillation of MN2L and MN2R during its late developmental stages (late tailbud to tail absorption) could be divided into seven phases (Phases I, II, III, IV, V, VI, VII) depending on the intervals and the synchronicity of MN2L and MN2R Ca^2+^ transients. Each phase is defined as follows (Figures 3B–H):

In Phase I, MN2L and MN2R oscillate sporadically (Figure 3B). Phase I was observed around St.22.

In Phase II, intervals of MN2L and MN2R bursts begin to converge to a constant value (-40 s) (Figure 3C). Phase II was observed from St. 22 to St.23.

In Phase III, MN2L and MN2R oscillate at constant intervals (Figure 3D). Phase III was observed around St.24.

In Phase IV, MN2L and MN2R oscillate at constant intervals (Figure 3E). Burst timing rarely synchronizes (Figure 3E, black arrowheads). This Phase could be considered as a transition Phase to MN2L and MN2R synchronization. Phase IV was observed around St.25.

In Phase V, burst timing of MN2L and MN2R synchronizes (Figure 3F) and oscillates at constant intervals. Phase V was observed from St.26 to St.28.

In Phase VI, burst timing of MN2L and MN2R synchronizes (Figure 3G) and oscillates at constant intervals longer than those of Phase V (Ex. Phase V intervals: 55 s → Phase VI intervals: 85 s). Long Ca^2+^ bursts (>30 s) were found to occur sporadically (Figure 3G, asterisks). Phase VI was observed from St.29 to St.30.

In Phase VII, the tail began to absorb (Figure 3H). Burst timing of MN2L and MN2R synchronizes. Intervals become sporadic. Long Ca^2+^ bursts (>30 s) were found to occur sporadically (Figure 3H, asterisks). Phase VII was observed after St.31.

A Rayleigh test of uniformity was conducted and circular plots for each phase were created (N = 5, Figure 4). It is notable that after Phase III, the R-value became larger (Figure 4) and the vector was around 0, indicating that after Phase III, Ca^2+^ transients of MN2L and MN2R were gradually becoming synchronized (more so than in the previous Phase II). In addition, a significant change was found in the R-values between Phase IV (0.391) and the later phases (>0.9 for Phases V, VI, VII), implying that the MN2L and MN2R Ca^2+^ bursts completely synchronize after Phase V.

### Relationships between tail movements and MN2 Ca^2+^ transients

**FIGURE 5:**
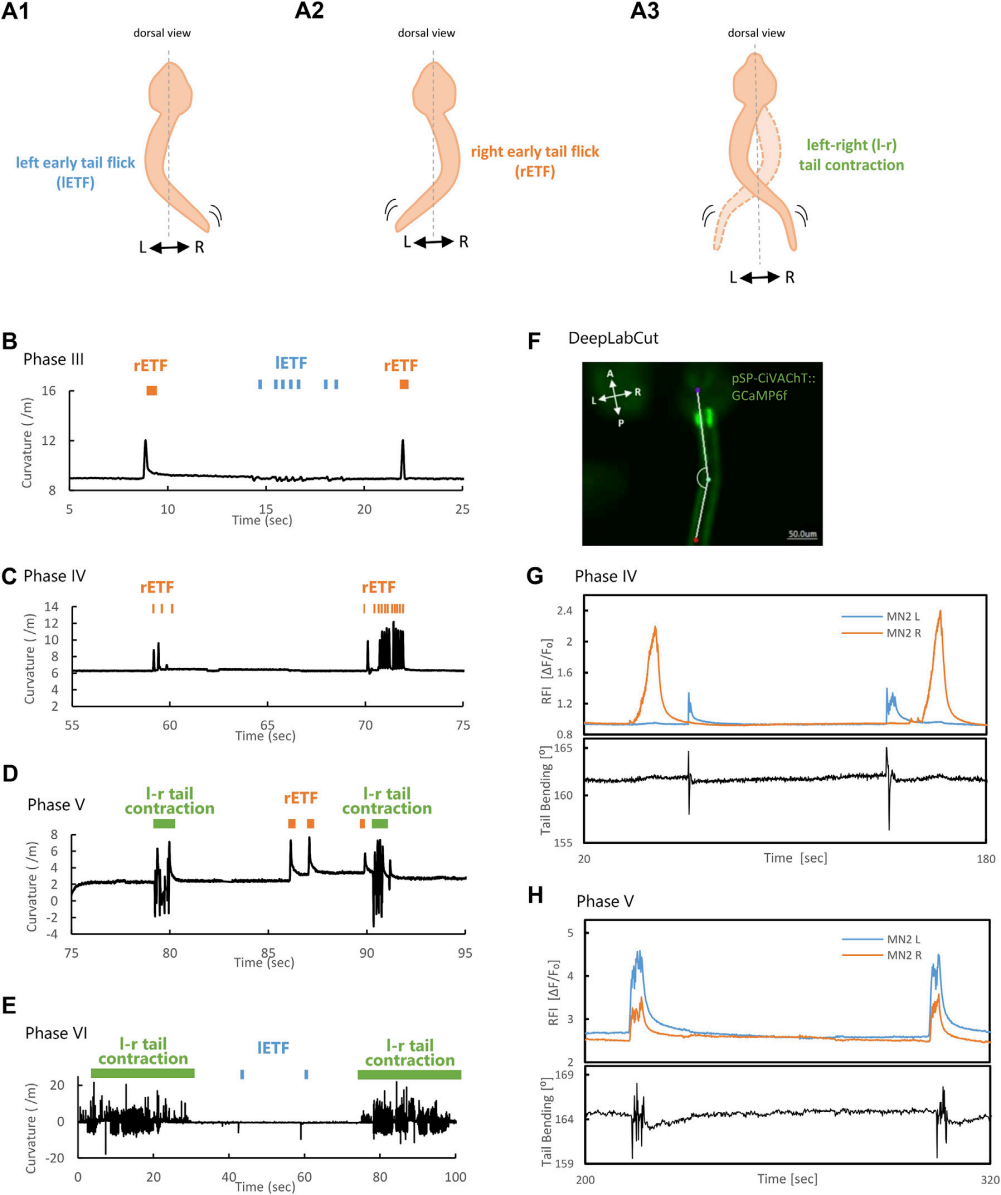
MN2L and MN2R Ca^2+^ transients and tail movement from Phase III to Phase VI. **(A)** Schematic illustration of left Early Tail Flick (lETF), right Early Tail Flick (rETF), and left-right (l-r) tail muscle contraction. **(B–D)** Tail curvature observed in each phase. Orange bar indicates right-sided Early Tail Flick, blue bar indicates left-sided Early Tail Flick, and green bar indicates continuous left-right alternate tail contraction (= swimming behavior). Longitudinal axis is curvature (/m); horizontal axis is time (sec). Time = 0 (sec) represents the timing when imaging started. Tail movement becomes faster in the later phases. **(B)** Phase Ⅲ. **(C)** Phase IV. **(D)** Phase V. **(E)** Phase ⅤI. **(F)** Image with three body parts (“bodypart1”, purple, ocellus; “bodypart2”, green, middle of the tail; “bodypart3”, red, terminal of the tail) tracked automatically with DeepLabCut. The original video was taken by a 3CCD camera (500 msec/frame). White lines are skeletons (a line connecting the three tracked body parts). **(G–H)** Tail bending and MN2 Ca^2+^ transients. Longitudinal axis is Relative Fluorescent Intensity (ΔF/F₀), and tail bending (°); horizontal axis is time (sec). Tail bending quantified with DeepLabCut. Time = 0 (sec) represents the timing when imaging started. **(G)** Phase IV, St.24 (late tailbud Ⅱ). Tail flicks correspond only with Ca^2+^ bursts seen in MN2L. **(H)** Phase V, St.27 (swimming larva). Left-right continuous tail muscle contractions are seen during the synchronous Ca^2+^ bursts of MN2L and MN2R.

To observe the relationships between tail muscle contractions and Ca^2+^ transients of MN2L and MN2R, tail movements were quantified by a high-speed camera from Phase III to Phase VI (N = 3). A spontaneous unilateral muscle contraction seen before the swimming larva stage is referred to as Early Tail Flicks (ETFs; (Akahoshi et al., 2021)) (Figures 5A1, 2). On the other hand, left-right alternate continuous tail muscle contractions (= swimming behaviors) were observed during the larval stage (Figure 5A3).

Before Phase V (before MN2L and MN2R Ca^2+^ transients completely synchronized), tail muscle contractions occurred only unilaterally (Figures 5B, C, orange and blue bars, “right-sided Early Tail Flick (rETF)”, “left-sided Early Tail Flick (lETF)”, respectively). This result clearly indicated that swimming behaviors did not occur before Phase V. However, during Phase V to Phase VI (Figures 5D,E), left-right continuous contractions of tail muscles (swimming behaviors) (Figures 5D, E, green bar, “l-r tail contraction”) were observed. In addition, tail muscle contraction’s frequencies in each phase were as follows: Phase III, 4.3 ± 0.38 Hz; Phase IV, 7.2 ± 0.71 Hz; Phase V, 9.7 ± 1.3 Hz; Phase VI, 10 ± 2.2 Hz. This suggests that the tail movement experienced a more than twofold increase in the later phases.

The duration of left-right continuous tail contracting behavior in Phase VI became significantly longer than that of Phase V (Phase V, -5 s; Phase VI, -30 s), suggesting that Phase VI larvae can continuously contract their tails and swim for a longer period than in Phase V. These tendencies correspond with the intervals and durations of MN2 activity captured by Ca^2+^ indicator GCaMP6s, suggesting that the Ca^2+^ transients captured with GCaMP6s were not tracking each single tail muscle contraction, but instead were capturing these ETFs and left-right continuous contractions as a single Ca^2+^ burst.

Due to the small K_off_ value of GCaMP6s (K_off_ = 1.1 (Chen et al., 2013)), the relationship between each muscle contraction and Ca^2+^ spikes in the swimming larva could not be fully explained. Therefore, by using a Ca^2+^ indicator with a larger K_off_, which enabled the indicator to capture a faster Ca^2+^ burst, we attempted to validate whether only the action potential, or also Ca^2+^ spikes are tuned to tail movements. To test this, a Ca^2+^ indicator with a larger K_off_, GCaMP6f (K_off_ = 3.9 (Chen et al., 2013)) was used to image faster Ca^2+^ transients in MN2.

Tail movements during the Ca^2+^ transients were tracked with DeepLabCut (Figure 5F). As a result, the Ca^2+^ bursts captured with GCaMP6f were notched (Figures 5G, H), especially in Phase V (Figure 5H), indicating that multiple firings may be occurring at MN2 during these single Ca^2+^ bursts in the earlier developmental stage (Phase IV, St.24, Figure 5G). In addition, tail movement synchronized only with MN2L Ca^2+^ burst and not with that of MN2R, meaning that the maturation of the neuromuscular junction could be asymmetric. In the later developmental stage (Phase V, St.27, swimming larva, Figure 5H), the synchronously occurring Ca^2+^ burst timings of MN2L and MN2R and the tail flicking movements synchronized. Overall, these results suggest that their swimming movements (left-right alternate tail muscle contractions) are seen during Phase V, at the timing of MN2L and MN2R’s synchronized Ca^2+^ elevation.

### Independent single-cell photostimulation of MN2L and MN2R with hChR2

In the previous study, Ca^2+^ oscillation coincided with Ca^2+^ elevation in the ipsilateral tail muscle; it was also shown that MN2 stimulation was necessary to evoke tail flicks in St.24 (Akahoshi et al., 2021). However, we could not simultaneously simulate MN2L or MN2R by single-cell photostimulation.

**FIGURE 6:**
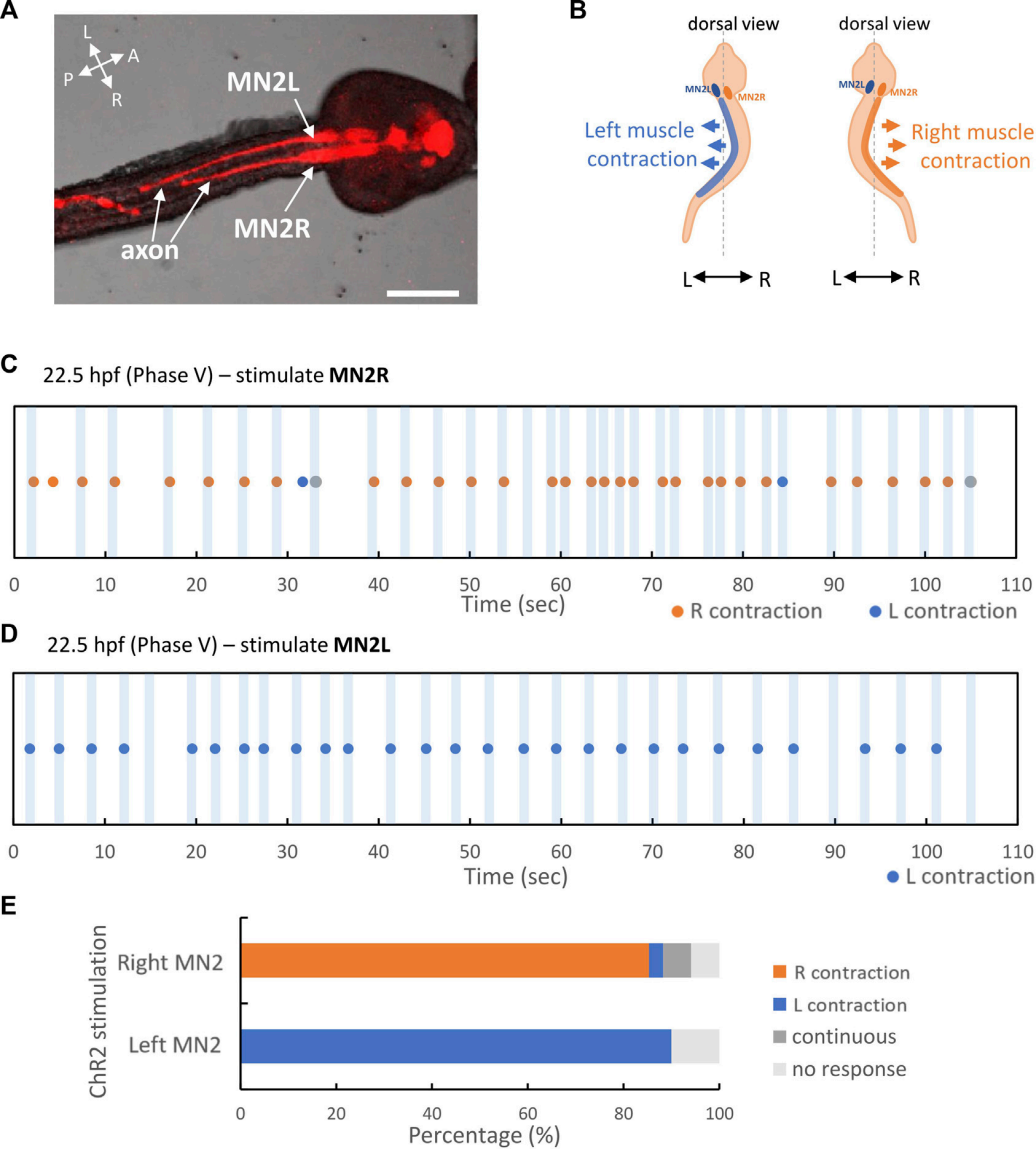
MN2L and MN2R regulate their unilateral muscles respectively. pSP-Neurog-hChR2(E123T/T159C):mCherry transduced swimming larva, photostimulated and imaged by CLSM. Larva was fixed on the dorsal side. MN2L and MN2R were irradiated independently. Orange dots indicate the time point when right tail muscle contracted, blue dots indicate the time point when left tail muscle contracted, and gray dots indicate the time point when tail flicked left and right continuously (swimming behavior). See also Supplementary Video S3. **(A)** Stacked confocal image of the stimulated larva. Images were taken by CLSM (DIC + fluorescent images with 594 nm excitation). Scale bar 50 µm. **(B)** Schematic illustration of right muscle contraction and left muscle contraction. **(C)** 22.5 hpf swimming larva, only MN2R stimulated. **(D)** 22.5 hpf swimming larva, only MN2L stimulated. **(E)** Percentage of behavioral responses (Left contraction, Right contraction, Continuous swimming, No response) during the laser stimulation in MN2L and MN2R. MN2R stimulation: Right contraction = 85%, Left contraction = 2.9%, Continuous swimming = 5.9%, No response = 5.9% (n = 34); MN2L stimulation: Left contraction = 90%, No response = 10% (n = 30).

In this study, *Ciona* embryos were fixed at the dorsal side; then hChR2-expressing MN2L and MN2R were independently stimulated by laser irradiation in St.27 swimming larva (N = 3, Figure 6A). Tail movement during MN2 stimulation was evaluated as to whether the left-sided muscle contracted or the right-sided muscle contracted (Figure 6B).

As a result, it was shown that MN2s are sufficient to contract ipsilateral tail muscle even after the swimming larva stage. When only MN2R was stimulated for a short period of time (-0.3 s), the tail flicked to the right side, meaning that the right-sided muscle contracted (Right contraction = 85%, Left contraction = 2.9%, Continuous swimming = 5.9%, No response = 5.9% (n = 34)) (Figures 6C, E). On the other hand, when only MN2L was stimulated for a short period of time (-0.3 s), the tail flicked to the left side, meaning that the right-sided muscle contracted (Left contraction = 90%, No response = 10% (n = 30)) (Figures 6D, E). This result indicates that MN2 regulates ipsilateral tail muscle contraction even in the larval stage (Supplementary Video S3).

## Discussion

**FIGURE 7:**
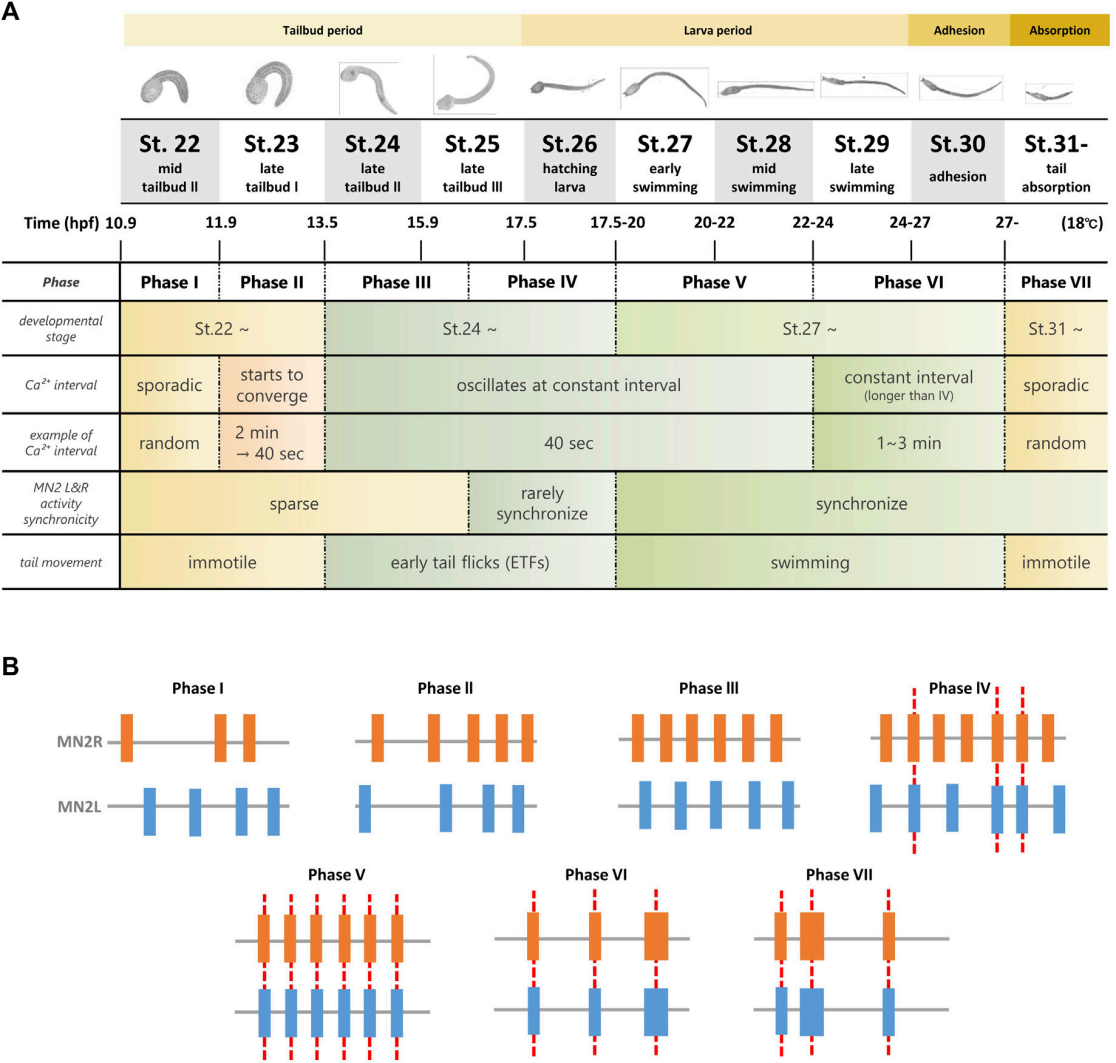
Summary of the seven Phases of MN2L and MN2R Ca^2+^ transients observed from tailbud stage to tail absorption. **(A)** Ca^2+^ transients were observed during tailbud, swimming larva, adhesion, and tail absorption periods (shown in yellow band). Long-term simultaneous Ca^2+^ imaging of MN2s with GCaMP6s/f (St.22-) revealed that their activities could be classified into seven phases (Phase I to Phase VII), depending on the interval and synchronicity of MN2L and MN2R Ca^2+^ transients. Initially, each MN2 oscillates sporadically (Phase I); as they develop into swimming larvae, they gradually oscillate at a constant interval (Phase II - Phase III), rarely synchronize (Phase IV), synchronize from the swimming larva period (Phase V), oscillate at a longer interval (Phase VI), and start to exhibit sporadic bursts during the tail absorption period (Phase VII). **(B)** Schematic illustration of Ca^2+^ transients from Phase I to Phase VII. Red dotted lines indicate synchronization of Ca^2+^ bursts between MN2L and MN2R.

This study revealed that MN2s are involved in the pattern generation of tail movements even after St.24, and their spontaneous Ca^2+^ oscillation continues to the tail absorption period. From the synchronization of MN2L and MN2R Ca^2+^ bursts, MN2s’ Ca^2+^ transition was divided into seven phases (Figure 7). Interestingly, axon outgrowth of MN2s was asymmetric, and 76% of the larvae started to oscillate from MN2R, which showed a faster axon extension rate than the other. Optogenetic experiments (single-cell photostimulation of MN2 by hChR2) supported the notion that each MN2 directly mediates its ipsilateral tail muscle contraction. However, the mechanisms of how they collaboratively contract their tail muscles left-right alternately during their swimming movement (Figure 5) is still unknown. We can discuss neural circuit development according to the seven phases of MN2 activities observed in this study as follows.

### Phases I, II, and III

In Phase I, Ca^2+^ transitions of MN2R and MN2L are completely sporadic. This implies that in the early developmental stages (around St.22), MN2R and MN2L work independently as a pattern generator for each of the ipsilateral muscles without any transmissions between these left and right neurons. However, as Ca^2+^ transitions change from Phase I to Phase III, intervals of MN2L and MN2R bursts converge to a constant value (-40 s). There was an individual difference in which one MN2 started to oscillate earlier than the other MN2, though MN2R had a higher tendency to be earlier than that MN2L (76%, N = 13/17). Entrainment starts from Phase III. (See the circular plot of the Rayleigh test of uniformity in Figure 4.) The possible factors working behind this MN2 Ca^2+^ oscillation entrainment are as follows:

1) As other contralateral neurons in *Ciona* MG (ex. ACIN1, ACIN2, ddN) mature, the contralateral axons connect MN2R with MN2L, reinforcing the entrainment of their Ca^2+^ oscillations, which eventually synchronize to the other MN2.
2) MN2s themselves contract their Ca^2+^ oscillations to a 40-s interval independently, without help from other neurons.

Future work is required to understand the mechanisms for this frequency convergence of MN2R and MN2L Ca^2+^ oscillation.

Meanwhile, the axon growing speed observed during Phases I to IV was asymmetric; axon outgrowth of the earlier oscillating motor neurons was faster than that of others (Figure 2; Supplementary Video S2). This implies that MN2 Ca^2+^ oscillation may be involved in its axon growth rate. Axon outgrowth requires axon guidance molecules, though such cues used for both MN2 and ACIN in *Ciona* remain unknown.

However, several axon guidance molecules, such as *Ephrin*, and *Semaphorin 3A*, have been primarily found in the notochord of *Ciona* tailbud embryos (Hotta et al., 2000; Kugler et al., 2008). *Ciona* also has a well conserved homolog of a vertebrate axon guidance molecule, *Netrin*. The vertebrate *Netrin* is expressed predominantly in the floor plate of their spinal cords, in the case of the *Ciona* tailbud embryo, *Netrin* is mainly expressed in their notochords, and also in a portion of the central nervous system (ddNs, descending decussating neurons) (Hotta et al., 2000; Gibboney et al., 2020). These molecules could also be working as guidance cues for MN2s.

### Phases III and IV

The significant difference between Phases III and IV is that in Phase IV, MN2L and MN2R Ca^2+^ bursts start to synchronize. Though this synchronization is intermittent, the change implies that information exchange has started between MN2L and MN2R to make either of them coincide with the other MN2’s burst timing.

### Phases IV and V

The significant difference between Phases IV and V is that from Phase V, MN2L and MN2R Ca^2+^ bursts start to synchronize permanently. The high-speed recording of the tail muscle contraction also reveals that Phase V belongs to the swimming larva stage, in which the somatic left and right tail muscles contract alternately and generate a continuous tail-beating ≒ swimming behavior (Figure 5H). As shown in the experiment that involved individually stimulating either MN2L or MN2R (Figure 6), unilateral activation of MN2 evokes only its ipsilateral behavior (muscle contraction on the ipsilateral side). From this hChR2 simulation, it was revealed that in order to evoke not only ipsilateral tail muscle contraction but also left-right alternate muscle contraction, both MN2L and MN2R might have to be activated coordinatively and reciprocally.

### Phases V and VI

The differences between Phases V and VI is that the Ca^2+^ burst intervals of Phase VI are longer than those of Phase V, and also that Phase VI larvae can continuously contract their tails and swim for a longer period of time than those in Phase V (Figure 5).

Recent research revealed that ascidian larvae exhibit multiple types of swimming, and their tail curvature varies depending on the stimulus. (Athira et al., 2022; McHenry, 2001; Nishino et al., 2011). Further analysis and fine tuning of DeepLabCut training data might be required to analyze the correlation between Ca^2+^ oscillations and these various types of swimming in detail.

It is known that *Ciona* swimming larva shows a behavioral sequence starting with a sporadic tail movement of negative geotaxis (causes the larva to swim upwards), followed by negative phototaxis (“shadow response”, causes the larva to swim downwards) (Zega et al., 2006; Meinertzhagen and Imai, 2008). These phototaxis behaviors are regulated by the upper neural circuit. *Ciona* larval brain contains two pigmented organs (the otolith and the ocellus), and 177 neurons. For instance, Groups I and II photoreceptors are responsible for phototaxis and gravitaxis, respectively, and activate swimming *via* pr-AMG RNs, which project to MGINs (Olivo et al., 2021). Phases V and VI may reflect such phototactic inputs from upper neural circuits as well as differences observed in swimming behavior, although further experiments are needed to fully understand the role of MN2s during swimming pattern generation during the swimming larva period.

### Phases VI and VII

In Phase VII, MN2 oscillates sporadically although it is under metamorphosis and its tail is absorbing. The tail becomes immotile in this developmental stage (Hotta et al., 2007), suggesting that the neuromuscular junctions between the motor neurons and the somatic muscles have been ablated. However, MN2L and MN2R are still synchronized, meaning that the connection between these two neurons that were required for the swimming behavior will not be ablated even after their tails stop beating. This may be showing the robustness of the MN2 oscillating swimming circuit.

### Half Center Model in *Ciona* swimming larva

When Brown’s Half Center Model is adapted to the *Ciona* motor circuit, it could be hypothesized that the Ca^2+^ activity of MN2R and MN2L is reciprocally driven by the receipt of contralateral inhibitory inputs from other neurons, such as ACIN. However, it was shown that the Ca^2+^ burst timings of MN2L and MN2R were synchronous in the swimming larvae, though their tail beating was left-right alternate. The possible reasons for this are as follows:

1) In this study, fluorescent images of Ca^2+^ transitions were obtained with a frame rate of 0.2- sec/frame. Asynchronous Ca^2+^ bursts of MN2L and MN2R could be recorded with a higher frame rate camera or by transducing Ca^2+^ sensors with a higher sensitivity.
2) In the later swimming larvae, muscle contraction may still be finely tuned by MN2 action potentials (as described in Akahoshi et al., 2021). In this case, an experiment to image MN2L and MN2R action potentials from their lateral side is required.

However, in this study, fluorescent images of Ca^2+^ transitions were obtained with a minimum frame rate of 0.2 s/frame. Realtime membrane potential imaging of both MN2s in future research is adequate to understand the coordination between them. Though Ca^2+^ bursts of MN2 were synchronized, each Ca^2+^ spike occurring inside one Ca^2+^ burst may not be synchronized when recorded with a higher frame rate or by transducing other sensors with a higher sensitivity.

## Data Availability

The original contributions presented in the study are included in the article/Supplementary Material, further inquiries can be directed to the corresponding author.
