# Supplementary material for: Transitions of motor neuron activities during Ciona development
Source: Front Cell Dev Biol. 2023 Jan 13;11:1100887. doi: 10.3389/fcell.2023.1100887 (PMC9880257; doi:10.3389/fcell.2023.1100887)
Supplement: Supplementary file 2 [file Table2.docx]

Supplementary Material

**Supplementary Tables**

**Supplementary Table 1. Statistical data of MN2L and MN2R Ca^2+^ bursts, intervals, durations, and synchronicity for each Phase (N=10, [A]-[J])**

Data for intervals, durations, Ca^2+^ Phase, developmental stages, synchronization, and bursts obtained from the long-term imaging of MN2L and MN2R’s Ca^2+^ oscillation with GCaMP6s transduced *Ciona* embryos (Trial [A] to [J] ,N = 10). Calculation methods for intervals and durations are based on Supplementary Fig 1. Average ± S.D. are shown for each interval (sec) and duration (sec). In the “synchronization” section, × indicates the Ca^2+^ oscillations of MN2R and MN2L are asynchronous, △ indicates the Ca^2+^ oscillations of MN2R and MN2L randomly synchronizes, and 〇 indicates the Ca^2+^ oscillations of MN2R and MN2L are permanently synchronized.

**Supplementary Figures**

**
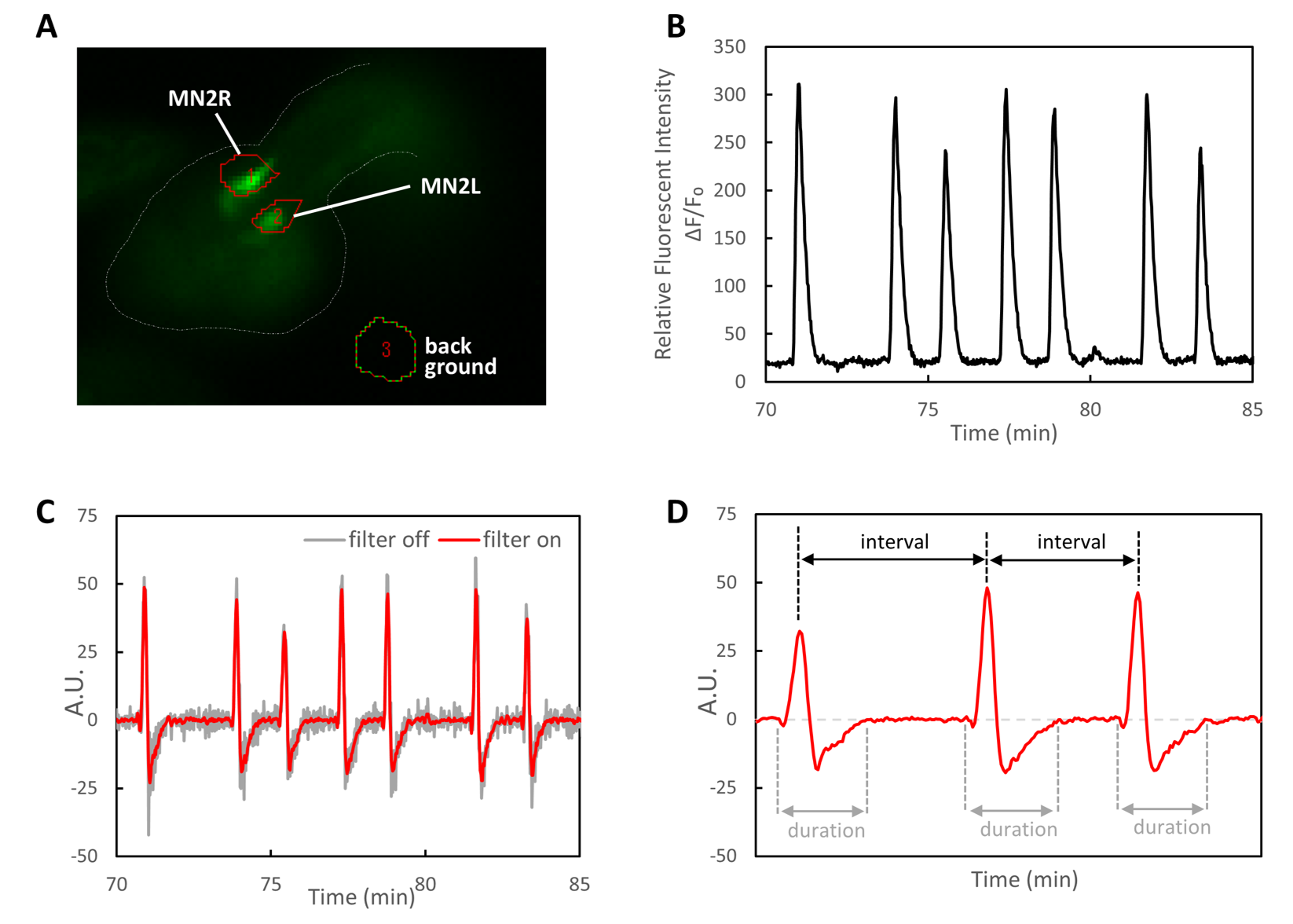
**

**Supplementary Figure 1. Definition of Ca^2+^ Burst peaks, burst intervals and durations.**

(A) Fluorescence microscopic view of *Ciona* larva. ROIs for MN2L, MN2R, and background are shown. (B) Temporal changes in Relative Fluorescent Intensity (RFI; ΔF/F₀). (C) RFI processed in MATLAB. Normalized, differentiated, and denoised with a Savitzky-Golay filter. Gray, signals before being filtered; red, signals after being filtered with a Savitzky-Golay filter. (D) Schematic illustration of the burst interval and duration of MN2 Ca^2+^ oscillation. Red, signals after being filtered with a Savitzky-Golay filter.

**Supplementary Videos**

**Supplementary Video 1. MN2L and MN2R Ca^2+^ transients from Phase I to Phase VII.**

Long-term imaging of MN2L and MN2R’s Ca^2+^ oscillation with pSP-VAChT::GCaMP6s transduced *Ciona* embryo, taken with fluorescent microscopy. Fixed to the glass based-petri dish at its dorsal side.

**Supplementary Video 2. Axon outgrowth of MN2L and MN2R.**

Long-term imaging of MN2L and MN2R’s Ca^2+^ oscillation with pSP-VAChT::GCaMP6s transduced *Ciona* embryo. Taken with CLSM. Fixed to the glass based-petri dish at its dorsal side.

**Supplementary Video 3. Single-cell photostimulation of MN2L and MN2R by hChR2.**

The movie is separated into two parts. First part: hChR2(E123T/T159C)-mCherry (corresponding to MN2) expressing MN2L stimulated by the 488-nm laser. Second part: hChR2(E123T/T159C)-mCherry expressing MN2R stimulated by the 488-nm laser. Duration for laser stimulation is 0.3 sec. The timing for photostimulation is indicated in the bottom left.
